# Gait Performance as an Indicator of Cognitive Deficit in Older People

**DOI:** 10.3390/ijerph18073428

**Published:** 2021-03-25

**Authors:** Juan Antonio Párraga-Montilla, Diana Patricia Pozuelo-Carrascosa, Juan Manuel Carmona-Torres, José Alberto Laredo-Aguilera, Ana Isabel Cobo-Cuenca, Pedro Ángel Latorre-Román

**Affiliations:** 1 Department of Didactics of Music, Plastic and Corporal Expression, University of Jaén, 23071 Jaén, Spain; jparraga@ujaen.es (J.A.P.-M.); platorre@ujaen.es (P.Á.L.-R.); 2 Department of Nursing, Physiotherapy and Occupational Therapy, Faculty of Physiotherapy and Nursing of Toledo, University of Castilla-La Mancha, 45005 Toledo, Spain; dianap.pozuelo@uclm.es (D.P.P.-C.); juanmanuel.carmona@uclm.es (J.M.C.-T.); anaisabel.cobo@uclm.es (A.I.C.-C.); 3 Multidisciplinary Research Group in Care (IMCU), University of Castilla-La Mancha, 45005 Toledo, Spain; 4 Social and Health Care Research Center (CESS), University of Castilla-La Mancha, 16071 Cuenca, Spain

**Keywords:** clinical evaluation, cognitive impairment, cognitive task, fitness, functional evaluation, gait performance

## Abstract

Background: The purpose of this study was to analyze which gait variables are the best for detecting cognitive impairment and to determine if age and gender can influence gait variations in older people. Methods: 65 participants took part in this study (22 men and 43 women; age: 73.88 ± 9.56 years). We use the Montreal Cognitive Assessment (MoCA) to assess mild cognitive impairment (MCI). Gait speed (GS) and the complex gait test (CGT) were analyzed with photocells Witty (Microgate, Italia). The OptoGait system (Microgate, Italia) was used to analyze step length (SL) and step coefficient of variation (CV sl). Results: There was a significant association between MoCA and SL (r = 0.420; *p* = 0.002), CV sl (r = −0.591; *p* < 0.001), and CGT (r = −0.406; *p* = 0.001). Instrumental activities of daily living showed significant association with SL (r = 0.563; *p* < 0.001); CV sl (r = −0.762; *p* < 0.001), CGT (r = −0.622; *p* < 0.001), and GS (r = 0.418; *p* < 0.001). CV sl showed the best results with MoCA when linear regression analysis was applied (R^2^ = 0.560; *p* = 0.007; Y = 23.669 − 0.320x). Participants older than 79 years showed lower MoCA scores and poorer gait parameters than people younger than 79 years. Conclusions: CV sl, SL, CGT, and GS make it possible to detect MCI in older people, especially when these variables are evaluated as a whole.

## 1. Introduction

Aging affects physical and cognitive functions [1]. During the aging process, there is a loss of capacities (physical and cognitive) compared to the younger people and to themselves with a younger age. This process affects walking performance and the cognitive involvement necessary to carry out the motor tasks of daily life [2]. Gait is considered an important indicator of general health status [3]; evaluating it allows professionals to predict or identify adverse cognitive episodes [4]. Gait is an automated rhythmic motor action that depends on a neuromechanical system directed by subcortical brain regions [5]. As age increases, there is a greater need to study the action of walking, as it requires greater dependence on cortical function, particularly the prefrontal cortex [6,7]. Therefore, older people must work harder to adapt to overcome environmental loads because greater cognitive involvement is required during the performance of a motor task [6,7]. Furthermore, poor gait performance is associated with higher levels of morbidity and mortality and a greater risk of falling [8]. It has been showed that as age increases, there is a deterioration in the functional reserve. It increases the sensitivity to external aggression that causes frailty, sarcopenia, falls, disability, and hospitalization and induces a deterioration in the quality of life and physical fitness [9,10].

Previous studies have established that there is an important relationship between motor performance and cognition [8,11,12]. Gait can be understood as a cognitive–motor sensory model, making it a highly complex skill [13]. This can be observed in many activities of daily life in which several tasks that demand greater attention resources are carried out at the same time. In fact, single gait (walking in a straight line without obstacles) requires an organization of motor control that is different from a complex or dual-task gait (walking while attending to different external stimuli). It is believed that higher-level control, which is necessary for complex gait, has a limited role in simple locomotion. There is little to no cortical participation during simple gait; this differs from complex situations that require inputs from higher-order frontal and parietal cortical regions to adapt gait to a new context [11,14]. These situations that require double-task or complex gait often exceed the capacity of the elderly. These abilities become more difficult when an individual has a cognitive problem or of an older age [15]. Therefore, to respond effectively in complex gait environments, an adequate capacity for the integration of external sensory information with cortical neuronal networks is required. These networks involve subcortical structures of the brainstem and spinal cord [16]. In this context, Hillel et al. [6] showed that gait parameters for people with neurodegenerative diseases and many other cohorts with walking disabilities were modified when they were subjected to double-task situations, often leading to a decrease in speed and an increase in variability and asymmetry. According to Plummer et al. [17], walking disabilities causes a significant limitation of mobility and an increased risk of falling when individuals are exposed to physical and/or cognitive contingencies with high interference while walking.

García Pinillos et al. [18] concluded that gait speed (GS) is an important predictor of mild cognitive impairment (MCI) in older adults, highly correlated with functional independence and comorbidity. This gait variable is dependent upon the difficulty of the task and the greater or lesser physical and/or cognitive involvement it requires; hence, a lower speed can help focus greater attention to complex cognitive tasks in order to improve performance [19]. For that reason, a decrease in GS has been observed in double-task situations that involve greater complexity, whereas the variability of the step (CV sl) increases in people with MCI [13]. Furthermore, the increase in stride time when performing dual tasks appears to be dependent on whether the task requires attention rather than speed [20]. Gillain et al. [21] concluded that GS and CV sl make it possible to identify mild cognitive impairments at an early stage and the likelihood of developing Alzheimer’s disease in the future. In particular, GS, CV sl, and step length (SL) are the most widely used measures to predict MCI and health status in older people [4,18,22,23]; when complex cognitive tasks are used, longer times are required for all tests [24].

In addition, different studies have shown that GS is an important variable to detect frailty and can predict the occurrence of adverse events [25]; speeds below 1 m·s^−1^ have been established as a reference point for episodes of frailty [26] and 0.8 m·s^−1^ has been considered a cut-off point in the evaluation of survival and life expectancy in older adults [27]. Therefore, the adverse effects of aging affect gait and cause a decrease in GS since it alters the step time, turning time, posture time, and double support time [28,29]. Important associations have been found between CV sl and an increased risk of falling. When GS, stride length, and the frequency, intensity, variability, smoothness, symmetry, and complexity of the gait decreases [30], there is also a decrease in strength, balance, functional status, and mental health [31].

The performance of motor tasks that demand attention and involve physical and cognitive capacity simultaneously is presented as a novel indicator for the detection of physical and cognitive frailty [15,32,33]. Hence, average performance during mobility and gait-related tasks has been used in numerous studies as a predictor of MCI and Alzheimer’s disease [4,23]. In this sense, the variability of different gait parameters is an important indicator of impaired executive function and movement control, as well as a predictor of an increased risk of falling [31]. Traditionally, gait has been evaluated under laboratory conditions (i.e., with structured and predictable tests for the participant) that have reported significant findings. The main limitation of these studies, however, is that the participants are isolated from their natural environment. There are differences in the results when the participants are evaluated in laboratory conditions and in the conditions of daily life where real conditions of motor action are required [6,34,35]. Currently, there is research focused on evaluating the brain regions that are structurally or functionally connected to each other as defined neural networks [36]. Therefore, an analysis from a multifactorial perspective can be a good gait assessment strategy as a predictor of adverse cognitive events [4].

To our knowledge, there are no studies that have compared the predictors of different gait variables or, more specifically, that quickly and accurately relate gait to functionality and cognition in older people in an ecological environment. Therefore, it is necessary to increase knowledge of predictive models of cognitive deterioration through the use of gait indicators that reproduce the activities of daily living with the greatest reliability. Previous research has assessed similar variables to those in our study [37,38]. This research was, however, conducted in a clinical environment. Consequently, the purpose of this study is to analyze which gait variables are best for detecting cognitive impairment and to determine if age and gender can influence gait variations in older people. Our initial hypothesis was that the relationship between cognitive impairment and gait performance should comprehensively take into account all of the descriptors that define gait performance.

## 2. Materials and Methods

### 2.1. Study Design and Participants

We conducted a cross-sectional study. A prior sample size was performed using The G*Power software [39]. The following parameters were selected for ANOVA (analysis of variance): large effect size (f = 0.500), α level of 0.05, a power level of 0.95, three groups, two covariates, critical F = 3.150, and a nonsphericity parameter λ = 16.250. The sample size was determined to be at least 65 participants.

A total of 65 people over 60 years of age (with an age range of 60 to 97 years) participated (mean age: 73.88 ± 9.56 years; median: 72.00 years), including 43 women (mean age: 73.37 ± 9.52 years; median: 71.00 years) and 22 men (mean age: 74.86 ± 9.77 years; median age: 74.00 years). Three age groups were established: 60–69 years, 70–79 years, and older than 79 years. Participants were recruited from four centers in southern Spain (Andalusia). To be included in the study, participants had to be (a) older than 65 years of age, (b) prosthesis-free, (c) free of any symptoms or exclusion criteria mentioned above after completing a medical examination performed by a professional team, (d) free of any pathologies associated with an increased risk of falling (e.g., Parkinson’s disease), (e) independently ambulatory, and (f) free of any disease that required daily medications that may affect gait performance (to avoid any influence on fitness measures). The exclusion criteria were (a) cardiovascular diseases such as ischemic heart diseases or stroke, (b) dementia or cognitive disease such as Parkinson’s disease, (c) problems with mobility, (d) inability to walk independently, (e) any other pathology that prevented them from moving without help, and (f) any cognitive disease that did not allow them to understand the protocol of evaluation. Each participant provided written informed consent for the study, which was carried out in accordance with the standards of the Declaration of Helsinki (2013 version). The study was approved by the Ethics Committee of the University of Jaén (Spain) (Protocol Code: OCT.20/7.PRY). 

### 2.2. Materials and Testing

Sociodemographic information including date of birth, marital status, education status, current occupational status, daily number of hours watching television, number of falls in the last three years, and days of physical activity per week were self-reported. To evaluate the participants’ complex activities of daily living and independent living skills, the Lawton Instrumental Activities of Daily Living Scale [40], Spanish version [41] was used. Regarding the internal consistency reliability, this test showed a Cronbach’s alpha = 0.94. The instrument contains eight items, with a summary score ranging from 0 (low function, dependent) to 8 (high function, independent) for women and 0 to 5 for men.

#### 2.2.1. Anthropometric Variables

Body mass (kg) was measured with a balance (Seca 899; Hamburg, Germany). Body height (cm) was measured with a stadiometer (Seca 222, Hamburg, Germany). Body mass index (BMI) was calculated by dividing the body mass (kg) by the square of the body height (m).

#### 2.2.2. Gait Variables

Gait speed (GS) over 5-m was measured with photocells (WITTY, Microgate Srl; Bolzano, Italy; 0.001-s accuracy) that were placed at the beginning and end of a 5-m corridor. The machines automatically recorded the time that participants took from the start point to the end point. The GS test involves walking 5-m in the shortest possible time [42].

We used the OptoGait System (Microgate Srl; Bolzano, Italy) to assess the variability of step length (CV sl) and step length (SL) in terms of the coefficient of variation (CV) among participants, given as a percentage *SD*/mean × 100% (% CV). OptoGait is an optical data acquisition system composed of a transmitter and a receiver bar. Each 1-m bar contains 96 infrared LEDs (1041 cm resolution) and is located on the transmitter bar, continuously communicating with the LEDs located on the receiver bar. The bars measure flight and contact times during execution with an accuracy of 1/1000 of a second. Regarding to the reliability of OptoGait System, all variables analyzed showed high concurrent validity with ICCs ranging between 0.933 and 0.999 (*p* < 0.001) (cycle time (s) = 0.999, stance time (s) = 0.983, swing time (s) = 0.933, step length (cm) = 0.997, cadence = (steps/min) = 0.999, and walking speed (m/s) = 0.999) [43].

The complex gait test (CGT) [44] was performed in an indoor controlled environment that simulated some of the features of social ambulation. The CGT has reported adequate reliability and validity parameters. In the test–retest analysis, the intra-class correlation coefficient was 0.868 (*p* < 0.001). There was a significant correlation between the CGT and trail-walking test (r = 0.592; *p* < 0.001).

Each participant was instructed to walk as fast as possible on a flat and straight 6-m path, turn right, weave between four posts, turn right again, walk by stepping alternately in a grid of five rectangles and high-step over two obstacles, turn right one last time, and walk 4-m to the finish line. The test score was the running time, with a longer time indicating a worse performance. The CGT was performed twice and the best trial was recorded. This test required six cones, four flags (1.5-m high), and masking tape to draw the grid.

#### 2.2.3. Cognitive Measures

The Montreal Cognitive Assessment (MoCA) [24] is the neuropsychological test recommended for MCI screening in older people [45]. The MoCA is a 30-point test that takes 10-min to complete and evaluates different aspects such as attention, concentration, orientation, calculation, language, verbal memory, recall, abstraction, visual–spatial ability, and executive function. A cut-off score < 21 indicates MCI [46]. The Spanish version was used [47]. The Cronbach alpha coefficient was 0.772.

### 2.3. Procedure

**Figure 1:**
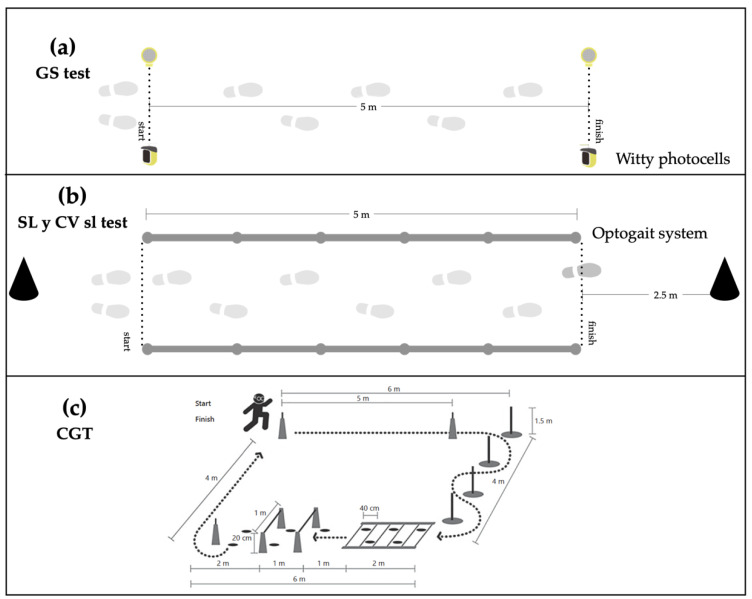
Assessment tests of gait performance variables. (**a**) Gait speed (GS), (**b**) stride length (SL) and gait variability (CV sl), and (**c**) complex gait test (CGT).

The evaluation was carried out in two separate sessions (48 h apart) by a team of researchers previously trained to evaluate the different tests. In the first session, the sociodemographic questionnaires were passed, which were completed individually. Researchers were present while the participants completed it and, they clarified any doubts or question (data confidentiality were respected at all times). Subsequently, we evaluated the anthropometric variables, the MoCA test, and the Lawton index. In a second session, the gait variables were performed (Figure 1). Before beginning the assessments, a familiarization with the three gait tests was conducted. After that, the participants performed each of the walking tests in the same order, with a 10-min break between each one. If the participants had to repeat the test, a total recovery between attempts was allowed (2 min approximately) in order to avoid muscular fatigue.

Participants performed the GS test first (Figure 1a). They started the test with one foot on the starting line in the front upright position. Counter time began when the subjects passed the first photocell and ended when the second photocell was crossed. Two attempts were made and the best one was recorded for analysis. A start signal was not provided so that the subjects could individually initiate the test. Therefore, the reaction time does not influence our findings. After that, we evaluated the SL and CV sl (Figure 1b). For that, a corridor made up of ten parallel bars of the OptoGait system (five transmitters and five receivers), which were placed in parallel on a 5-m long walkway, was used. The subjects began the test with one foot on the starting line located 2.5-m before the first OptoGait bar. To avoid acceleration and deceleration during the test, the participants walked 2.5-m before and after the 5-m corridor. We only recorded the 5 m corresponding to the OptoGait system. The subjects walked in their own shoes and the walking speed was selected by themselves. The only instruction was “walk at your normal speed”. In total, 60-m of walking were recorded. It corresponded to crossing 12 times along the 5-m corridor. Finally, the CGT test (Figure 1c) was performed. The participants had to complete the circuit in the shortest possible time. During the test, it was not possible to run and they were encouraged to do the test by walking as fast as possible. The participants started the test freely. Counter time was activated when the first photoelectric cell was crossed and it stopped when the subject cross the second photoelectric cell placed at the end of the circuit. If the circuit was not performed correctly, it could be repeated.

### 2.4. Statistical Analysis

Data were analyzed using SPSS v.19.0 for Windows (SPSS Inc.; Chicago, IL, USA). The significance level was set at *p* < 0.05. Descriptive data were reported in terms of means, *SD*, and percentages. Normal distribution (Kolmogorov–Smirnov) and homogeneity (Levene) tests were conducted on all data before analysis. Differences between sex and age groups were analyzed using ANCOVA (analysis of covariance), after correcting gait measures for age and body height. Differences between age groups were analyzed using analysis of variance (ANOVA) adjusted by the Bonferroni test. To verify the relationship between MoCA parameters with BMI, fall number, Lawton index, and gait performance, a partial correlation analysis and a simple linear regression analysis (adjusted by age) were used [48]. Pearson correlation analysis was conducted between gait variables with the MoCa and other variables such as the Lawton index or the number of falls. The gait performance threshold that best discriminated MCI was determined by using the receiver-operating characteristic (ROC) curve. Binary logistic regression was performed using MCI as the dependent variable and gait performance as the independent variable.

## 3. Results

The sociodemographic characteristics of the participants by sex and age groups are shown in Table 1. There were no differences based on sex or age, nevertheless, we found differences based on age (*p* < 0.001) in instrumental activities of daily living (Lawton test). The group older than 79 years of age had worse scores than the other two age groups.

Table 2 shows the results of the participants as they corresponded to the gait variables according to sex and age groups. There were no significant differences according to sex. When comparing the different age groups, significant differences (*p* < 0.001) were observed in all the variables. The group older than 79 years of age had worse results in all variables than the other two age groups.

The Pearson correlation analysis showed significant correlations between the MoCA score and SL (r = 0.420, *p* = 0.002), CV sl (r = −0.592, *p* < 0.001), CGT (r = −0.408, *p* = 0.001), BMI (r = −0.349, *p* = 0.005), and Lawton index (r = 0.699, *p* < 0.001). Moreover, the Lawton index showed significant correlations with SL (r = 0.553, *p* < 0.001), CV sl (r = −0.746, *p* < 0.001), GS (r = 0.417, *p* = 0.005), and CGT (r = −0.594, *p* < 0.001). The number of falls displayed a significant correlation with BMI (r = 0.283, *p* = 0.044) and GS (r = −0.308, *p* = 0.031). Age showed a significant correlation with Lawton index (r = −0.504, *p* < 0.001), MoCA score (r = −0.477, *p* < 0.001), SL (r = −0.584, *p* < 0.001), CV sl (r = 0.646, *p* < 0.001), GS (r = −0.419, *p* = 0.001), and CGT (r = 0.622, *p* < 0.001).

Simple linear regression analysis revealed that SL, CV sl, and CGT were the variables of gait performance that showed association with MoCa score (Table 3).

**Figure 2:**
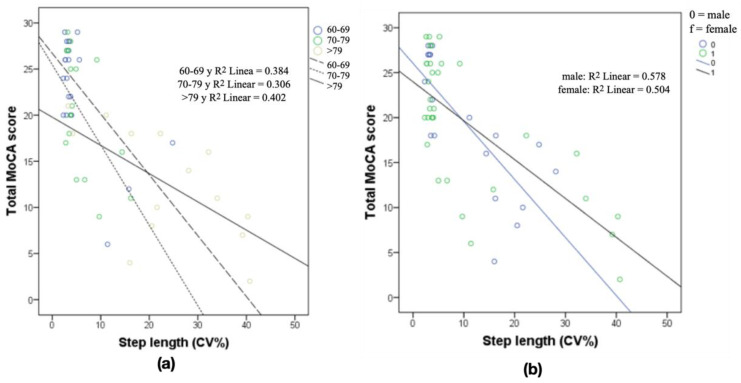
(**a**) Simple regression analysis relating CV step length and MoCA score in the three age groups and (**b**) CV step length and MoCA score in relation to the sex of the participants. R^2^, Pearson correlation.

Figure 2 shows the regression analysis between the MoCA test and the CV sl, adjusted to the three age groups and sex. The CV sl shows moderate to important predictive values of cognitive impairment by age, with a higher predictive level in the group older than 79 years of age and a similar level of prediction based on sex.

**Figure 3:**
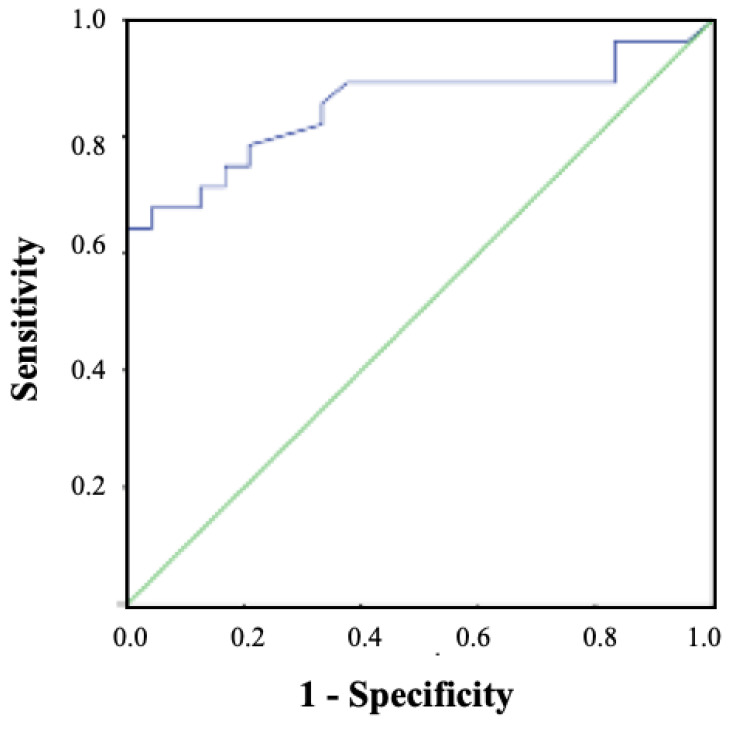
Receiver-operating characteristic (ROC)curve for mild cognitive impairment (MCI) prediction with CV sl (%) performance.

The multiple linear regression analysis showed that the CV sl (%) was associated only with the MoCA (R^2^ = 0.560; *p* = 0.007; Y = 23.669−0.320X). The binary logistic regression analysis revealed that a high CV sl (%) score was a risk factor for MCI (odds ratio = 1.757, 95% CI = 1.071–2.883; *p* = 0.026). Figure 3 shows the ROC curve for MCI prediction with CV sl (%) performance (area under the curve (AUC) = 0.852, 95% CI = 0.742–0.962; *p* < 0.001). The cut point was 3.9 s (sensitivity = 0.786, 1-specificity = 0.208).

## 4. Discussion

The purpose of this study was to analyze which gait variables are best for detecting cognitive impairment and to determine if age and gender can influence gait variations. Regarding the first purpose, the main findings of the current study show that all the gait variables analyzed are acceptable predictors of MCI, obtaining the highest associations in CV sl. Previous studies have concluded that GS, SL, CGT, and CV sl are the most widely-used measures to predict MCI and health status in older people [4,18,22,23,44]. The findings of the current study are consistent with those of Ijmkes et al. [49], who found that a decrease in executive function plays an important role in the increased variability of gait in patients with dementia, because the gait pattern becomes increasingly unstable in tasks with greater cognitive involvement. This confirms the idea that the older a person is, the more they will have to modify their gait pattern as the cognitive complexity of the task performed increases. This situation causes a reduction in speed, a shortened stride length, and a significant increase in variability during gait. These changes are associated with a protective mechanism against adverse events [18,25,50]. In this regard, slower temporal events and greater variability during walking have been reported for people at high risk of developing dementia, thus compromising survival and life expectancy in older adults [27].

Moreover, Klotzbier [24] shows that the greater the cognitive load directed at executive functions, the longer the time required to complete the complex gait test; thus, walking safely requires intact cognition and good executive control [27]. This may explain the increased risk of falling in older people who experience adverse cognitive events when the task exceeds the threshold of their cognitive capacity [8,17,29]. Previous studies have found important associations between gait variability and greater instability among older people in correlation with functional status and mental health, which allows for the detection of Alzheimer’s for those who are at risk [30,31]. Our data show a significant correlation between the number of falls with GS and BMI, and MoCa with BMI and with Lawton index.

It is also possible to assume that healthy older adults make their own adaptations to walk more efficiently and manage high cognitive demands. Postural control should be the first adjustment priority [2]; hence, the significant variation in SL may be evidence of a decrease in cognitive and/or physical capacity, presenting itself as a predictor of adverse events. In this sense, previous studies have shown that performance in double-task situations decreases with age, especially in the presence of cognitive impairment, and has been used as a potential instrument to evaluate the interaction between physical and cognitive functions [15,51]. Similarly, gait variability is strongly associated with cognitive function, as it affects brain changes associated with age, time, and coordination, both in terms of gait and cognition [46]. In fact, intra-individual variability in speed-related performance aging in older people has been proven to be a strong predictor of cognitive decline, brain aging, and increased risk of neurodegenerative disease. Since intra-individual variability occurs especially during neuropsychological tasks [52], it explains why gait speed decreases and variability increases in dual-task or complex gait situations [20]. To sum up, as previously discussed and in agreement with Latorre et., al [44], we consider walking performance to be a strong biomarker of health and of cognitive functioning in older people.

A second objective was to examine the influence of age and sex on gait performance. Overall, there are several age-related changes in gait performance. The oldest group (older than 79 years) obtained the worst results in all of the gait variables analyzed when compared with the other two age groups. These results match those observed in earlier studies [4,7,53] which indicated that gait performance parameters such as gait speed, gait variability, and walk with obstacle negotiation, change dramatically with age, which in turn show a significant association with cognitive impairment. In this regard, there is a close relationship between older age and higher levels of physical and/or cognitive impairment [15], in particular, the age of 80 years is a sensitive stage at which there is a greater risk of suffering physical and cognitive deficits [54].

Concerning sex, no significant differences between sexes for any gait parameters were found. According to [55], most of the spatio-temporal sex differences could be related to body size and self-selection of GS. However, when controlling for size, sex differences disappeared. In this regard, [56] highlight that there are no significant differences between sexes for any kinematics parameters of gait such as cadence, step length, step time, gait variability, and GS. Likewise, García Pinillos et al., [18] indicated that GS was similar in men and women. However, [57] showed that women walked with higher cadence (*p* = 0.01) and shorter stride length (*p* = 0.006) compared to men, while gait speed was not significantly related to sex. Therefore, more research on this topic needs to be undertaken.

The MoCA test has been shown to be an accurate instrument that makes it possible to compare gait and cognitive impairment [58,59] with the ability to reflect the state of gait in patients with cognitive impairment more accurately than other cognitive assessment instruments. When the time spent in performing dual-task walking is increased, a lower score in MoCA is achieved, which means that there is a greater predisposition to suffer from MCI [24]. As a consequence, the evaluation of the variables that define gait performance by simulating a real-life environment is a valid and reliable clinical marker for the detection of cognitive deficits in elderly and very old people [6].

Some limitations of this study should be mentioned. First, this was a cross-sectional study, so a longitudinal evaluation of the different variables is necessary. Second, the sample included Caucasian Spanish citizens, and a generalization to a wider population should be done with caution.

Despite the limitations indicated, the main strength of our study is the combined use of gait variables that allowed for an optimal prediction of MCI decline in elderly people. It is important to note that the tests we performed are easy to carry out and evaluate.

### Practical Applications

In the current study was incorporated the CGT in our evaluation, as it implies a greater cognitive motor commitment compared to walking in a straight line. Therefore, an evaluation of all the variables analyzed was presented as a good strategy for the detection and prediction of MCI in older people. It is a simple and inexpensive walking test in which CV sl, GS, SL, and CGT are evaluated in a comprehensive manner, allowing relevant information on the cognitive state of the elderly to be obtained. Therefore, and according to Kikker et al. [22], measures of walking capacity could assist as additional markers to predict cognitive decline. We recommend gait analysis, including several gait parameters, in clinical evaluations of patients with suspected cognitive decline. Later, qualified personnel will be able to continue the exploration if necessary.

## 5. Conclusions

In conclusion, certain parameters of human gait (especially CV sl) have an adequate capacity to predict cognitive impairment and functionality in older people, particularly when these are evaluated as a whole. Age, but not sex, is a factor that conditions these relationships. Therefore, CV sl, SL, CGT, and GS make it possible to predict and/or detect cognitive impairment. However, these variables are more effective when they are evaluated together. This comprehensive evaluation can be used as an early cognitive impairment indicator and to design and implement specific training programs for those who demonstrate gait abnormalities.

## Figures and Tables

**Table 1:** General characteristics of the participants divided by sex and age groups.

Variable	*n*	Total Mean (*SD*)	*n*	Men Mean (*SD*)	*n*	Women Mean (*SD*)	*p*-Valor	*n*	60–69 Years Mean (*SD*) a	*n*	70–79 Years Mean (*SD*) b	*n*	>79 Years Mean (*SD*) c	*p*-Valor	Post Hoc Analysis
Age (years)	65	73.88 (9.56)	22	74.86 (9.77)	43	73.37 (9.52)	0.556	25	65.08 (3.12)	25	73.24 (2.36)	16	88.06 (5.19)	<0.001	
BMI (kg/m^2^)	63	26.98 (3.44)	21	27.51 (3.43)	42	26.71 (3.46)	0.394	23	26.31 (3.29)	25	26.96 (3.36)	15	28.04 (3.77)	0.325	
TV (hour/day)	51	2.89 (1.52)	18	3.02 (1.33)	33	2.81 (1.62)	0.643	19	2.76 (1.41)	16	3.37 (1.14)	16	2.56 (1.89)	0.292	
PA (day/week)	52	4.00 (2.15)	18	3.94 (2.31)	34	4.03 (2.09)	0.894	19	4.47 (1.83)	17	4.35 (1.93)	16	3.06 (2.51)	0.109	
Falls (number)	52	0.73 (1.15)	17	0.47 (1.00)	35	0.86 (1.21)	0.262	19	0.58 (1.01)	17	0.65 (0.86)	16	1.00 (1.54)	0.536	
Lawton index (point *)	48	6.00 (2.53)	17	5.436 (2.45)	31	6.26 (2.58)	0.346	18	7.00 (1.87)	15	7.13 (1.72)	15	3.67 (2.41)	<0.001	a < c ***; b < c ***

BMI, body mass index; PA, physical activity; TV, hours of television per day; Falls, number of falls in the last three years; and Lawton index, Lawton test for the evaluation of instrumental activities of daily life. Point *: female = 0–8, male = 0–5; a, 60–69 years; b, 70–79 years; c, >79 years, *** *p* < 0.001.

**Table 2:** Results of gait variables and the Montreal Cognitive Assessment (MoCA) test according to sex and age groups.

Variable	*n*	Total Mean (*SD*)	*n*	Men Mean (*SD*)	*n*	Women Mean (*SD*)	*p*-Valor	*n*	60–69 Years Mean (*SD*) a	*n*	70–79 Years Mean (*SD*) b	*n*	>79 Years Mean (*SD*) c	*n*	*p*-Valor	Post Hoc Analysis
SL (cm)	52	61.82 (11.13)	19	63.33 (13.68)	33	60.95 (9.49)	0.350	21	67.61 (8.88)	17	64.30 (8.67)	14	50.15 (7.95)	52	<0.001	a > c ***; b > c ***
CV sl (%)	50	10.44 (11.07)	19	10.64 (8.71)	33	10.32 (12.36)	0.247	21	5.34 (5.52)	17	5.94 (4.07)	14	23.55 (12.67)	52	<0.001	a > c ***; b > c ***
GS (m·s^−1^)	61	1.12 (0.52)	20	1.10 (0.59)	41	1.12 (0.49)	0.222	21	1.23 (0.53)	24	1.30 (0.52)	16	0.70 (0.22)	61	<0.001	a > c *; b > c **
CGT (s)	65	21.64 (8.48)	22	21.41 (8.66)	43	21.76 (8.50)	0.332	24	18.00 (5.87)	25	18.84 (5.27)	16	31.47 (8.52)	65	<0.001	a < c ***; b < c ***
MoCA (0–30 point)	65	(19.43 (7.23)	22	19.50 (7.24)	43	19.40 (7.30)	0.709	24	22.58 (5.96)	25	20.66 (6.55)	16	12.81 (5.87)	65	<0.001	a > c ***; b > c ***

SL: Step length; CV sl: Step length, step coefficient of variability; GS: Gait speed; CGT: Complex Gait Test. MoCA: The Montreal Cognitive Assessment; a, 60–69 years; b, 70–79 years; c, >79 years; *** *p* < 0.001; ** *p* < 0.01; * *p* < 0.05.

**Table 3:** Standardized beta coefficients from linear regression models on the associations of age, sex, anthropometric measures, Lawton index, number of falls, and performance in gait tests with MoCA score.

	MoCa		
	Beta	*p*-Valor	R^2^
Sex	−0.006	0.961	<0.001
Age (years)	−0.477	<0.001	0.228
BMI (Kg/m^2^)	−0.312	0.005	0.332
Lawton index (0–8)	0.697	<0.001	0.621
Number of falls	−0.212	0.084	0.301
SL (cm)	0.446	0.002	0.386
CV sl (%)	−0.669	<0.001	0.516
GS (m·s^−1^)	0.069	0.592	0.217
CGT (s)	−0.458	0.001	0.357

BMI, body mass index; Lawton index, Lawton test for the evaluation of instrumental activities of daily life; SL, step length; CV sl, step coefficient of variability; GS, gait speed; CGT, complex gait test; and MoCA, Montreal Cognitive Assessment; R^2^, Pearson correlation.

## Data Availability

Proyecto Andared. Financed by the CSIC foundation.

## Abbreviations

BMI	Body mass index
CGT	Complex gait test
CV	Coefficient of variation
CV sl	Step coefficient of variability
GS	Gait speed
MCI	Mild cognitive impairment
MoCA	Montreal Cognitive Assessment
SL	Step length
